# Survival correlation of immune response in human cancers

**DOI:** 10.18632/oncotarget.27360

**Published:** 2019-12-03

**Authors:** Yuexin Liu

**Affiliations:** ^1^Department of Bioinformatics and Computational Biology, The University of Texas MD Anderson Cancer Center, Houston, Texas, USA

**Keywords:** overall survival, progression-free interval, immune response, immunogenicity, cancer

## Abstract

Background: The clinical benefit of immune response is largely unknown. We systematically explored the correlation of immune response with patient outcome in human cancers.

Results: The global immune gene signature was primarily located on the plasma membrane with a high gene density at 6p21 and 1q23-1q24. Immune responses varied with a wide range in human cancers. A total of 11 cancer types exhibited significant correlation of immune response with overall survival. Higher immune response was significantly associated with longer overall survival in 7 types and with shorter overall survival in 4 types. In addition, 11 cancer types exhibited significant correlation of immune response with progression-free interval. Higher immune response was significantly associated with longer progression-free interval in 7 types and with shorter progression-free interval in 4 types.

Methods: The Ingenuity Knowledge Base and human genome assembly GRCh38 were used to annotate the immune gene signature by cellular components and genomic coordinates, respectively. We devised an mRNA-based metric of pre-existing immune conditions by using the gene signature, and calculated the metric for 10,062 The Cancer Genome Atlas tumor samples across 32 different cancer types. The Kaplan-Meier method was used to evaluate the overall survival and progression-free interval differences between dichotomic groups stratified by the median metric for each cancer type.

Conclusions: Immune responses have different impacts on patient outcome in different human cancers. Prospective verification is needed before the findings can be applied for clinical trial development.

## INTRODUCTION

Development of immunotherapy such as checkpoint blockade therapy (against CTLA4 or PD-1/PD-L1) has shown great success in potentiating inefficient antitumor immune response and inducing durable control of tumors [1, 2], thus leading to improved patient survival in different types of cancer [3, 4]. However, the efficacy or clinical benefit of immunotherapy has been demonstrated to intimately depend upon the pre-existing immune conditions of a tumor [5–7]. It was previously reported that high expression of chemokines in the tumor microenvironment promoted T cell infiltration and induced immune-mediated tumor regression [8, 9]. IFN-γ produced by T helper type 1 cells was shown to activate cytotoxic T cells and potentiate the cell-mediated immune response in the tumor [10]. High pretreatment expression of PD-L1 protein was significantly associated with the anti-PD-L1 antibody [5], and pre-existing CD8^+^ T cells were significantly associated with therapeutic PD-1 blockade [7]. In addition, metastatic melanoma patients with overexpressed immune genes were more likely to respond favorably to ipilimumab (an antibody specific to CTLA4), and therefore had better clinical survival [6]. Our group recently demonstrated in endometrial cancer that genes negatively associated with survival were significantly enriched in immune-related pathways, while positively associated genes were predominantly cell cycle-related [11]. Consistent with that study were previous publications showing that high expression of immune gene signatures was associated with favorable prognosis in breast [12] and colorectal cancer [13]. Taking together, it appears that the pre-existing immune-active tumor microenvironment might favor clinical response to immunotherapy in some cancer types.

However, the clinical benefit of immune response is largely unknown in many other cancer types, and the relationship between immune response and patient outcome has not been systematically investigated in a wide array of human cancers. In addition, it remains a challenge to characterize the immune response of a tumor in a way that is scalable and comparable among different cancer types. Using an unbiased approach, we recently analyzed data for 10,062 tumor samples in The Cancer Genome Atlas (TCGA) PanCanAtlas data set to identify a global immune gene expression signature and demonstrated with multifaceted evidence that this signature of 382 immune genes is robust and broadly applicable for human cancers [14]. In contrast to other PanCanAtlas pathway members such as TGF-β [15] and DNA damage repair pathways [16] that were selected by the domain experts, the global immune gene signature [14] was identified through an unsupervised clustering analysis on an encompassing immune-related gene list. It was a data-driven result from a large pool of functionally defined immune genes and therefore was unbiased and comprehensive. In the current study, we have extended this work by using the same cohort of patients and the identified immune gene signature to devise an mRNA-based metric for pre-existing immune conditions, and we applied this metric to infer the clinical benefits of immune response in human cancers. We correlated this metric with patient outcomes, including overall survival (OS) and progression-free interval (PFI), in order to determine whether immune response varies across tumor types, whether immune response has the same direction of impact on clinical outcome for different cancer types, and whether immune response has the same impact on OS and PFI for individual cancer types. Further, the spatial characteristics of the immune genes in the signature including both cellular and chromosomal locations is also examined.

## RESULTS

### Spatial characteristics of the global immune gene signature

**Figure 1:**
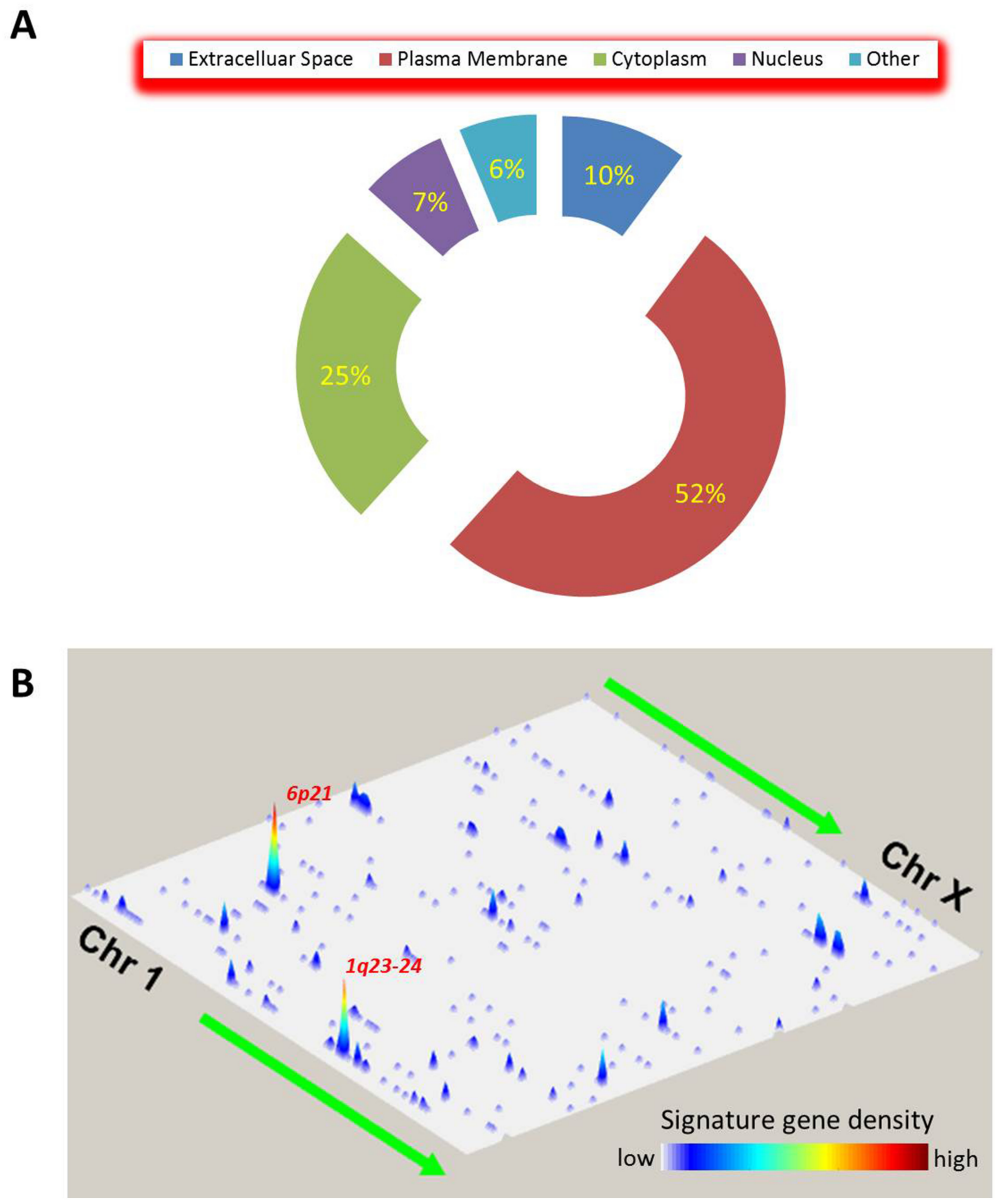
Spatial characteristics of the global immune gene signature. (**A**) Distribution of the immune signature genes among different cellular locations. The numbers indicate the percentage of the member genes in each category. (**B**) Distribution of the immune signature genes across genomic locations. The signature genes’ densities are plotted in two-dimensional space representing chromosomal positions of the human genome assembly GRCh38. One dimension consists of the 23 chromosomes from Chr 1 to Chr X; the other, the locations on a chromosome from short (*p*) arm to long (*q*) arm. The gene density is indicated by the peak height and color bar. The two genomic locations with the highest gene density are also shown.

To better understand the spatial characteristics of our previously identified global immune gene signature [14], we annotated the member genes’ cellular locations using the Ingenuity Knowledge Base. We found that the majority of the members of this gene set (~52%; 197 genes) were located on the plasma membrane, and only 7% (27 genes) were in the nucleus (Figure 1A). This cellular distribution makes sense because (i) the immune gene signature is indicative of the relationship between tumor cells and infiltrating immune cells, and (ii) the bipolar plasma membrane serves as an intimate interface between the cell and the surrounding tumor microenvironment. In addition, the genes on the plasma membrane are potent targets for therapeutic intervention, compared to those in the other cellular locations. Indeed, assessment of availability of targeting drugs showed that 59 of the 197 plasma membrane genes, but only 1 of the 27 nuclear members, were potential therapeutic targets.

Next, we examined the genomic coordinates of the immune gene signature in the human genome. Using a similar approach as described previously [17], we constructed the genome “landscape” of the immune gene signature in which the genes were plotted in two dimensions and the density at each region was indicated by the height of the peak (Figure 1B). We found that the signature’s genes were unevenly distributed across the genome, with heterogeneous peak heights. The p21 region on chromosome 6 (*6p21*) had the highest signature gene density, followed by the two consecutive cytoband regions on chromosome 1 (*1q23-24*).

### An immune metric derived from the immune gene signature

**Figure 2:**
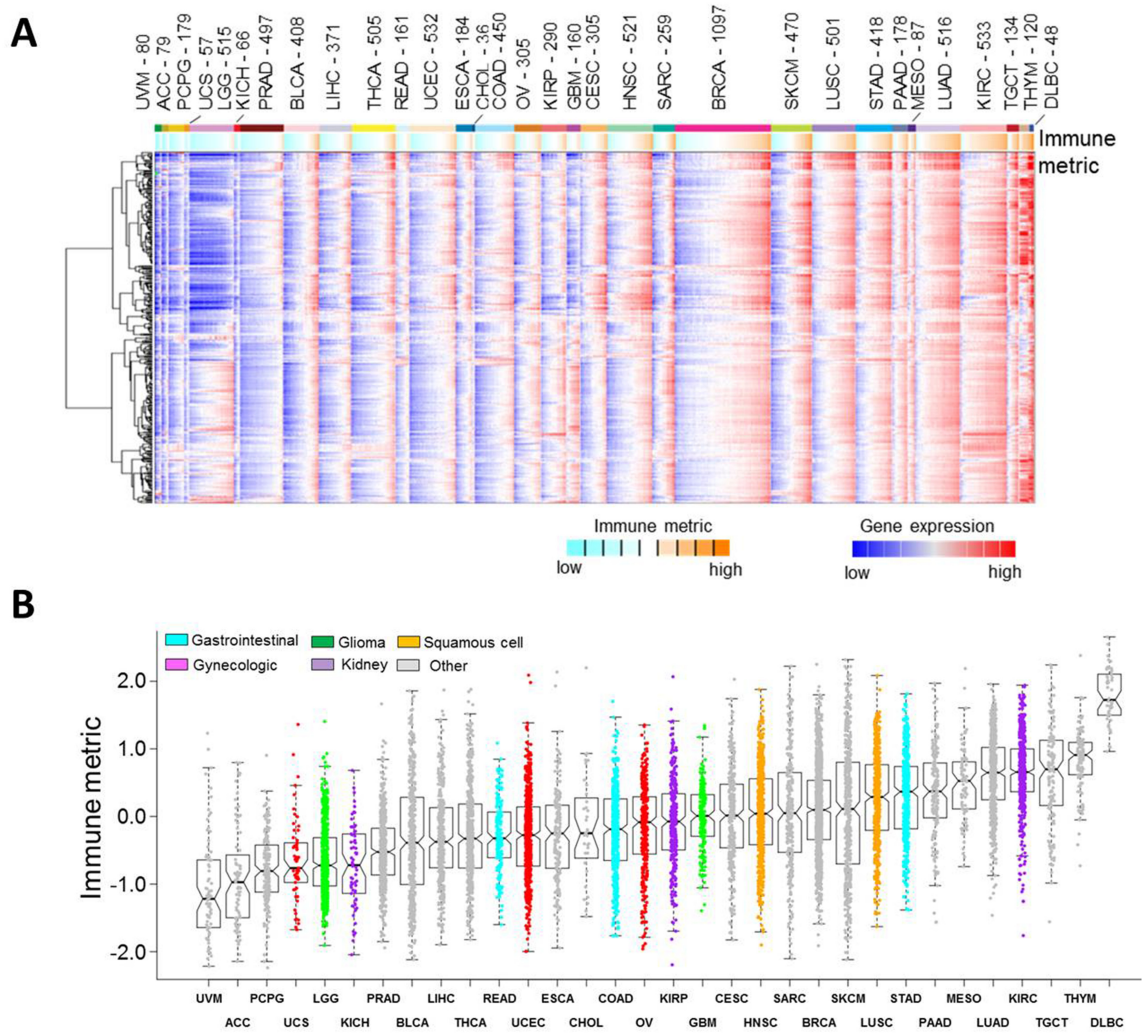
Immune response in human cancers. (**A**) Heat map of 382 member genes included in the immune gene signature across 10,062 PanCanAtlas tumors. Red, higher expression (values normalized to SDs from the median across all cancers); blue, lower expression. mRNA features were summarized into an immune metric for each tumor profile (orange, higher inferred immune response; cyan, lower immune response). Cancer types (denoted by TCGA project name) are ordered by low to high median immune metric. (**B**) Boxplots of immune response, as inferred using the transcript levels of the immune gene signature. From the bottom, the horizontal lines in the boxplots represent 5%, 25% (lower edge of box), 50% (center of box), 75% (upper edge of box), and 95%. Cells of origin are indicated by different colors. Tumor types with two possible origins, such as bladder urothelial carcinoma (squamous cell or other), cervical squamous cell carcinoma and endocervical adenocarcinoma (gynecologic or squamous cell), and esophageal carcinoma (squamous cell or other), are shown in gray. Abbreviations: ACC: Adrenocortical carcinoma; BLCA: bladder urothelial carcinoma; BRCA: breast invasive carcinoma; CESC: cervical squamous cell carcinoma and endocervical adenocarcinoma; CHOL: cholangiocarcinoma; COAD: colon adenocarcinoma; DLBC: lymphoid neoplasm diffuse large B-cell lymphoma; ESCA: esophageal carcinoma; GBM: glioblastoma multiforme; HNSC: head and neck squamous cell carcinoma; KICH: kidney chromophobe; KIRC: kidney renal clear cell carcinoma; KIRP: kidney renal papillary cell carcinoma; LGG: brain lower grade glioma; LIHC: liver hepatocellular carcinoma; LUAD: lung adenocarcinoma; LUSC: lung squamous cell carcinoma; MESO: mesothelioma; OV: ovarian serous cystadenocarcinoma; PAAD: pancreatic adenocarcinoma; PCPG: pheochromocytoma and paraganglioma; PRAD: prostate adenocarcinoma; READ: rectum adenocarcinoma; SARC: sarcoma; SKCM: skin cutaneous melanoma; STAD: stomach adenocarcinoma; TGCT: testicular germ cell tumors; THCA: thyroid carcinoma; THYM: thymoma; UCEC: uterine corpus endometrial carcinoma; UCS: uterine carcinosarcoma; UVM: uveal melanoma.

We previously defined a global immune gene signature that is capable of qualitatively characterizing tumor immunogenicity in a wide array of human cancers [14]. To quantitatively determine the immune response of the tumors in the TCGA dataset, in this study we generated an immune metric by taking the median expression of the 382 genes that were included in our previously identified immune gene signature [14]. A similar approach has successfully been used in prior studies from our group [15, 18] and others [19, 20]. The immune metric serves as a surrogate for tumor immune response, characterizing the pre-existing immune conditions of a heterogeneous tumor. For each tumor, the expression pattern of the 382 member genes included in the immune gene signature was shown and summarized into an immune metric (Figure 2A). On average, immune metrics differed by tumor lineage, with uveal melanoma (UVM) and adrenocortical carcinoma (ACC) tumors showing the lowest levels of immune response and diffuse large B-cell lymphoma (DLBC) showing the highest levels, followed by thymoma (THYM) and testicular germ cell tumor (TGCT). Consistent with our result, adrenocortical carcinoma was previously shown to have a low immune score due to the suppression of T cell activity by glucocorticoids [21]. Lung adenocarcinoma (LUAD) and skin cutaneous melanoma (SKCM) exhibited high immune response in our study, likely due to high mutational loads [22]. However, bladder urothelial carcinoma (BLCA), which was previously reported to have a relatively high mutational load [23], was shown in this study to have low immune response. Different from bladder urothelial carcinoma, thymoma has a relatively low mutational load [23] but was shown in this study to have high immune response, likely because thymoma is associated with autoimmune disorders [24]. Mesothelioma was previously reported to have a low tumor mutation burden [25], and our study showed that MESO had relatively high immune response and likely responded to immune-checkpoint blockade therapy [26, 27]. This observation is consistent with our recent work in which two groups of endometrial cancer patients had similar mutation burden but exhibited different immune profiles [11]. Taking these data together, we believe that the immune metric is a reliable quantification of immune response in human cancers, which does not appear to be fully explained by mutational loads. In addition, a wide range of immune responses were evident within each tumor type (Figure 2B). Immune response is largely dependent on a wide variety of regulatory factors, such as mutations, transcription factors, and microRNAs. MiRNA-34a was previously reported to regulate PD-L1 gene expression and modulate the immune response in acute myeloid leukemia [28]. *CTNNB1* gene mutation has been shown to be associated with low immune response [29, 30]. On the other hand, patients with *POLE* gene mutations had higher immune activity [31] and were associated with favorable prognosis compared with patients without *POLE* mutations [32]. These complicated regulatory networks have contributed, at least in part, to the wide spectra of immune response within an individual cancer type or across different cancer types. This may also explain the difference in the response rates to immunotherapy among patients even within the same cancer types.

### Association of immune response with patient overall survival

**Figure 3:**
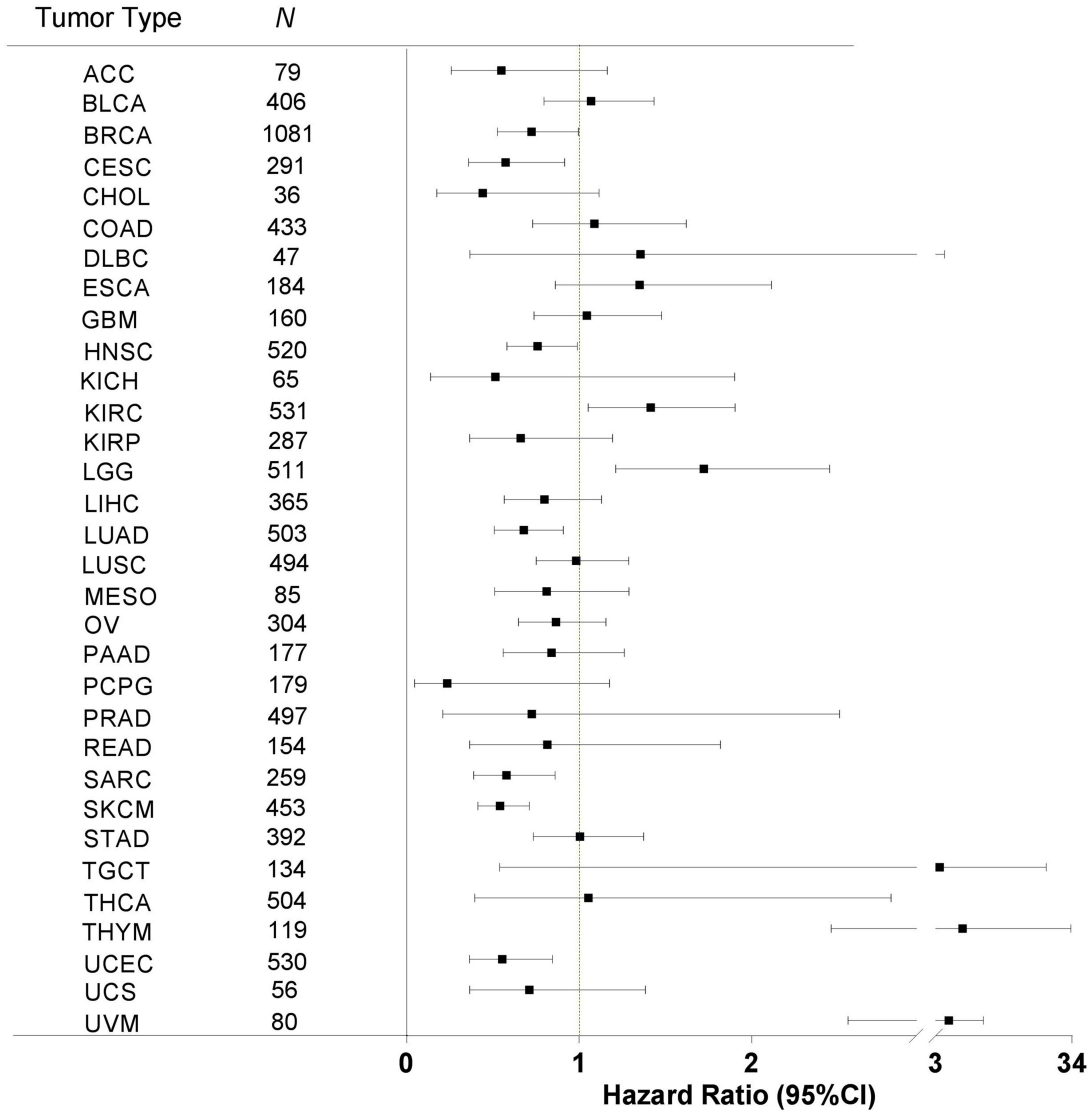
Immune response correlation with patient overall survival in human cancers. For each of the 32 cancer types, we correlated the tumor immune response with patient OS by using the Kaplan-Meier method with the Mantel-Cox log-rank test. The hazard ratio (HR, indicated by the solid square in the plot) and the corresponding 95% confidence interval (CI, indicated by the two vertical bars at the ends of the line) are shown. An HR value of less than 1 indicates that higher immune response is correlated with longer survival, and vice versa. The statistical significance depends upon whether the 95% CI range contains the value of 1. Tumor types are sorted in alphabetical order, and *N* indicates the number of analyzed samples.

Next, we sought to examine the relationship between immune response and patient OS. For each of the 32 cancer types, we performed Kaplan-Meier survival analysis of dichotomic groups, high and low immune metric, as determined by the median value, and then calculated the hazard ratio (HR) for the high versus low groups and the corresponding 95% confidence intervals (CIs) (Figure 3). As shown in Figure 3, 20 cancer types had an HR value of less than 1, meaning that these cancer types had an association of higher immune response metric with longer OS. The other 12 cancer types had an HR value of greater than 1, meaning that these cancer types had an association of higher immune response with shorter OS. A wide range of CIs were observed among cancer types. The 95% CI ranges in 11 cancer types did not include the value of 1, meaning these cancer types had a significant association of immune response with OS.

**Figure 4:**
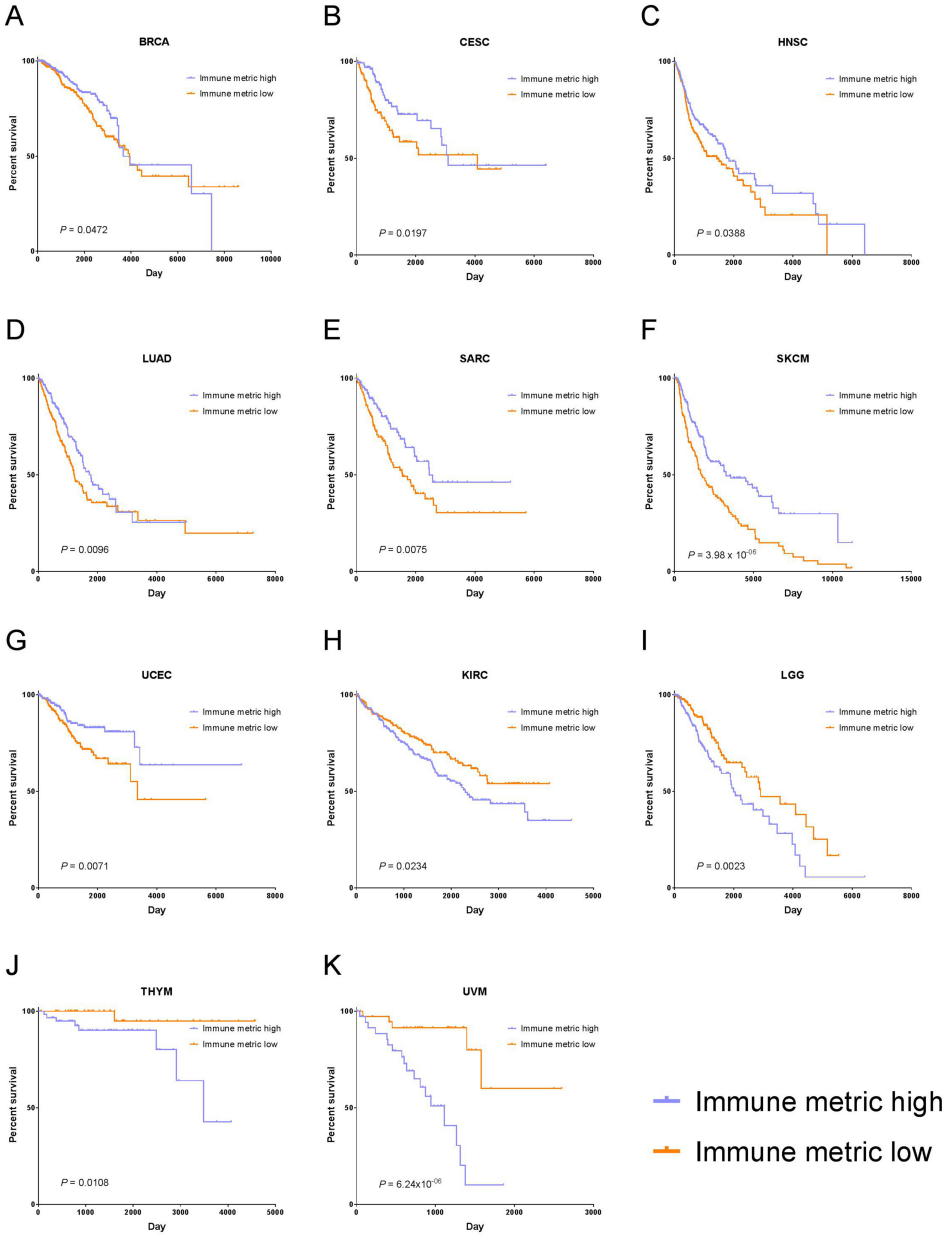
Tumor types with significant correlation of tumor immune response with OS. Kaplan-Meier survival curves for (**A**) Breast Invasive Carcinoma (BRCA), (**B**) Cervical Squamous Cell Carcinoma and Endocervical Adenocarcinoma (CESC), (**C**) Head and Neck Squamous Cell Carcinoma (HNSC), (**D**) Lung Adenocarcinoma (LUAD), (**E**) Sarcoma (SARC), (**F**) Skin Cutaneous Melanoma (SKCM), (**G**) Uterine Corpus Endometrial Carcinoma (UCEC), (**H**) Kidney Renal Clear Cell Carcinoma (KIRC), (**I**) Brain Lower Grade Glioma (LGG), (**J**) Thymoma (THYM), (**K**) Uveal Melanoma (UVM). In panels (A–G), higher immune response was significantly correlated with longer OS, and in panels (H–K), higher immune response was significantly associated with shorter OS.

In particular, higher immune responses were significantly correlated with longer OS in seven cancer types: breast invasive carcinoma (BRCA, *P* = 0.0472), cervical squamous cell carcinoma and endocervical adenocarcinoma (CESC, *P* = 0.0197), head and neck squamous cell carcinoma (HNSC, *P* = 0.0388), lung adenocarcinoma (LUAD, *P* = 0.0096), sarcoma (SARC, *P* = 0.0075), skin cutaneous melanoma (SKCM, *P* = 3.98 × 10^–06^), and uterine corpus endometrial carcinoma (UCEC, *P* = 0.0071) (Figure 4). In contrast, higher immune responses were significantly correlated with shorter OS in four cancer types: kidney renal clear cell carcinoma (KIRC, *P* = 0.0234), low-grade glioma (LGG, *P* = 0.0023), thymoma (*P* = 0.0108), and uveal melanoma (*P* = 6.24 × 10^–06^) (Figure 4). Aside from the cancer types mentioned above, the rest of the cancer types did not show significant correlation between immune response and patient OS.

### Association of immune response with patient progression-free interval

**Figure 5:**
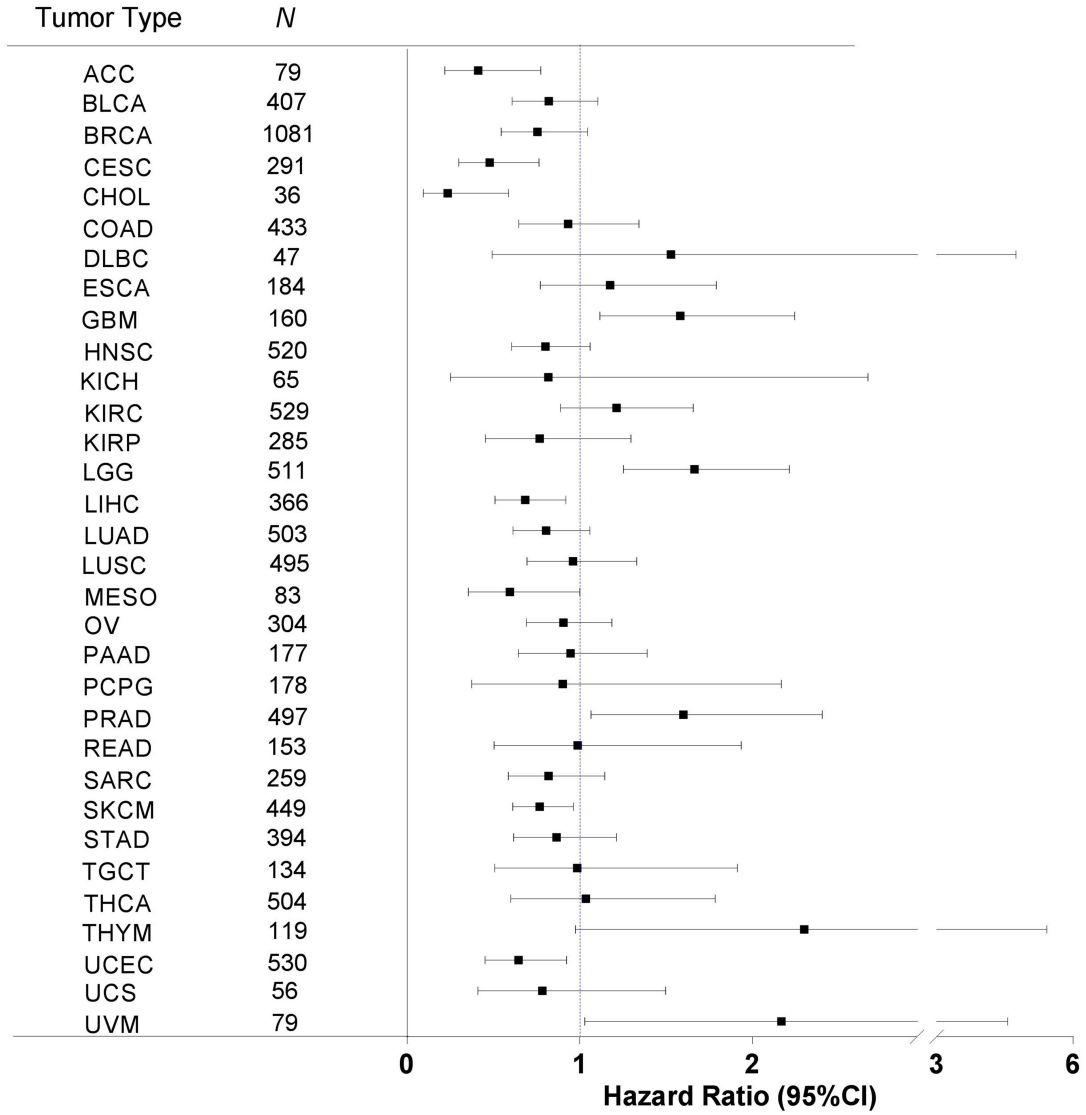
Immune response correlation with patient progression-free interval in human cancers. For each of the 32 cancer types, we correlated the tumor immune response with patient PFI by using the Kaplan-Meier method with the Mantel-Cox log-rank test. HRs (indicated by the solid squares in the plot) and the corresponding 95% CIs (indicated by the two vertical bars at the ends of the line) are shown. Tumor types (denoted by TCGA project name) are sorted in alphabetical order, and *N* indicates the number of analyzed samples.

In addition to OS, PFI provides an additional perspective of tumor progression and metastasis. We next interrogated the association of immune response with PFI in a similar manner as we performed the Kaplan-Meier method on OS. A total of 23 cancer types had an HR value of less than 1, meaning that in these cancer types, a higher immune response was associated with longer PFI. The other 9 cancer types had an HR value of greater than 1, meaning that in these cancer types a higher immune response was associated with shorter PFI (Figure 5).

**Figure 6:**
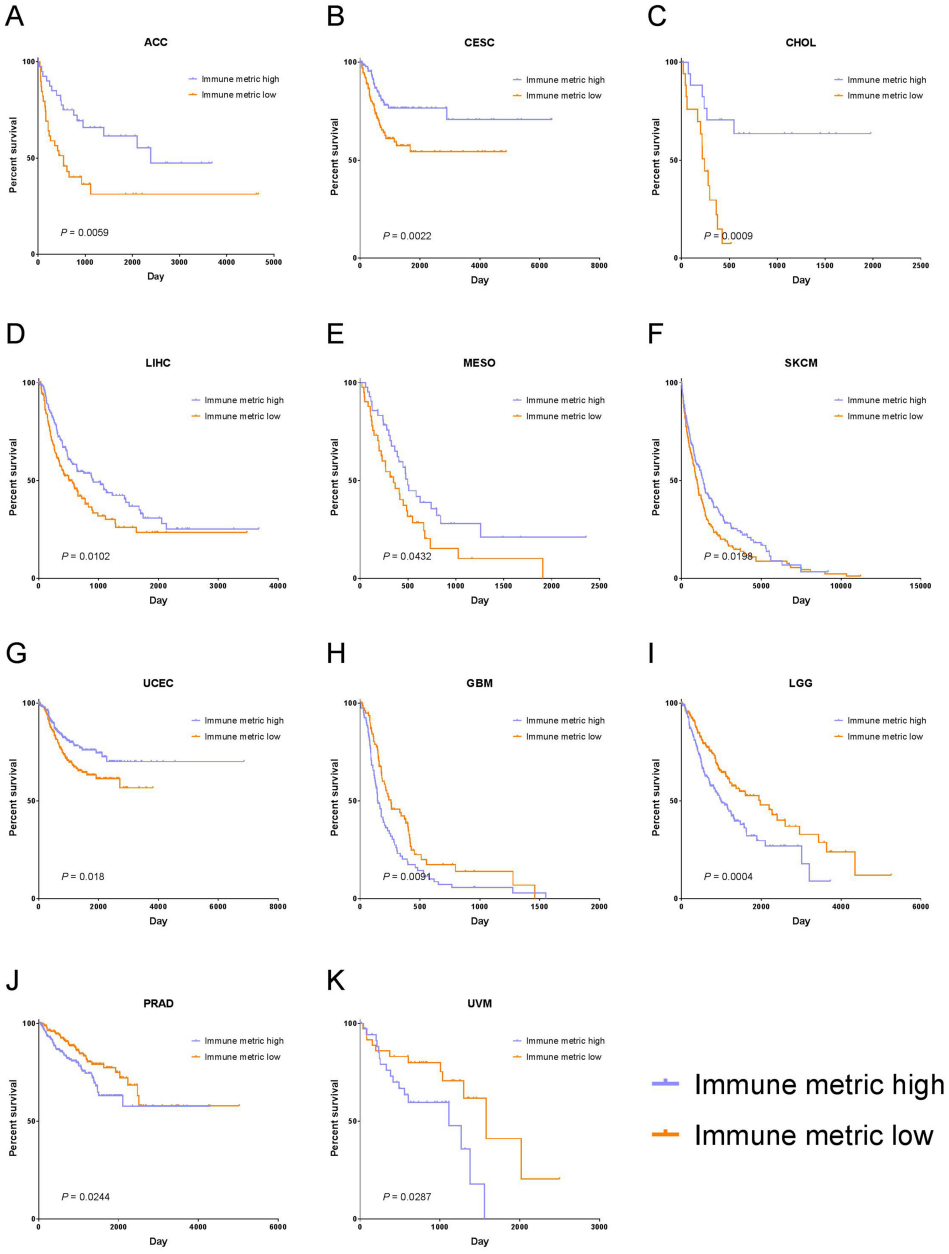
Tumor types with significant correlation of tumor immune response with PFI. Kaplan-Meier survival curves for (**A**) Adrenocortical carcinoma (ACC), (**B**) Cervical Squamous Cell Carcinoma and Endocervical Adenocarcinoma (CESC), (**C**) Cholangiocarcinoma (CHOL), (**D**) Liver Hepatocellular Carcinoma (LIHC), (**E**) Mesothelioma (MESO), (**F**) Skin Cutaneous Melanoma (SKCM), (**G**) Uterine Corpus Endometrial Carcinoma (UCEC), (**H**) Glioblastoma Multiforme (GBM), (**I**) Brain Lower Grade Glioma (LGG), (**J**) Prostate Adenocarcinoma (PRAD), (**K**) Uveal Melanoma (UVM). In panels (A–G), higher immune response was significantly correlated with longer PFI, and in panels (H–K), higher immune response was significantly associated with shorter PFI.

Also shown in Figure 5 is that 11 cancer types had a significant association of immune response with PFI, as evidenced by their corresponding 95% CIs. In particular, 7 cancer types—adrenocortical carcinoma (ACC, *P* = 0.0059), cervical squamous cell carcinoma and endocervical adenocarcinoma (CESC, *P* = 0.0022), cholangiocarcinoma (CHOL, *P* = 0.0009), liver hepatocellular carcinoma (LIHC, *P* = 0.0102), mesothelioma (MESO, *P* = 0.0432), skin cutaneous melanoma (SKCM, *P* = 0.0198), and uterine corpus endometrial carcinoma (UCEC, *P* = 0.018)—demonstrated a significant correlation of higher immune response with longer PFI. The other 4 cancer types—glioblastoma multiforme (GBM, *P* = 0.0091), brain lower grade glioma (LGG, *P* = 0.0004), prostate adenocarcinoma (PRAD, *P* = 0.0244), and uveal melanoma (UVM, *P* = 0.0287)—demonstrated a significant correlation of higher immune response with shorter PFI (Figure 6). The rest of the cancer types did not show significant correlation between the immune response and PFI.

## DISCUSSION

In this study, we assessed more deeply our recently identified immune gene signature that is applicable for all human cancers [14]. We identified the cellular and chromosomal locations of the 382 genes in the gene signature. We also devised an mRNA-based metric assessing pre-existing immune conditions in the tumor microenvironment and found that the metric varies with a wide range in human cancers. Our results provide a comprehensive view of the relationship between immune response and clinical outcome in human cancers.

Correlation with clinical outcome showed that in 7 cancer types (BRCA, CESC, HNSC, LUAD, SARC, SKCM, and UCEC), higher immune response was associated with significantly longer OS, and the opposite was the case for KIRC, LGG, THYM, and UVM. Consistent with these results, our recent work in endometrial cancer demonstrated that genes whose higher expression was associated with better survival were significantly enriched in immune-related pathways [11], and pre-existing immune condition was previously reported to favor the clinical outcome in skin cutaneous melanoma [5, 7]. Cervical cancer cases with lower immune response exhibited worse prognosis in our study, likely due to higher potentiality of epithelial-mesenchymal transition [33]. As compared with uveal melanoma tumors with D3 (disomy 3), those with M3 (monosomy 3) exhibited significantly worse prognosis but had significantly higher CD8 T cell infiltrates and significantly higher expression of genes involved in immune-related functions [34], which is consistent with our results. Moreover, a previous report consistently showed that kidney renal clear cell carcinoma with higher immune response was associated with worse survival [35]. Different from our study, a previous publication showed that a subset of thymoma patients with activated T-cell signaling exhibited a favorable prognosis [36].

In addition, ACC, CESC, CHOL, LIHC, MESO, SKCM, and UCEC demonstrated a significant correlation of higher immune response with longer PFI, opposite to the correlative patterns observed for GBM, LGG, PRAD, and UVM. The association of the immune response with PFI of glioma patients (including brain lower grade glioma and glioblastoma multiforme) is consistent with a previous report [37], demonstrating that activated microglia/macrophages in the tumor microenvironment promote glioma cell growth and invasion [38, 39]. Our results showed that liver hepatocellular carcinoma with higher immune response was not associated with OS, which is consistent with a previous publication [40]. However, these patients had significantly longer PFI and thus would likely respond to immune checkpoint inhibitor therapies. The immune response also had a significant and positive association with PFI in adrenocortical carcinoma. Consistent with these results, a previous report showed that adrenocortical carcinoma patients with enrichment of upregulated immune genes had significantly longer survival [21]. Although the difference was not significant, adrenocortical carcinoma also had well-separated OS curves in our study (data not shown). Similarly, cholangiocarcinoma exhibited significant association of immune response with PFI but not with OS although the OS curves were well separated.

We found that spatially, the 382 genes of the global immune gene signature [14] are clustered together with a high density in the regions of 6p21 and 1q23-24. The short arm of chromosome 6 contains the human leukocyte antigen (HLA) complex that encodes the major histocompatibility complex (MHC) proteins and is responsible for the regulation of the immune system in humans. MHC class I molecules, including HLA-A, -B, and -C, present peptides from inside the cell to T lymphocytes, while MHC class II molecules (HLA-DP, -DM, -DO, -DQ, and -DR) present antigens from outside the cell. The long arm of chromosome 1 contains several Fc receptor-like glycoproteins. In particular, the protein encoded by the *FCRL2* gene has four extracellular C2-type immunoglobulin domains, a transmembrane domain, and a cytoplasmic domain that contains one immunoreceptor-tyrosine activation motif and two immunoreceptor-tyrosine inhibitory motifs. FCRL2 expression was previously reported as a prognostic factor in chronic lymphocytic leukemia [41, 42].

Our results show that in the majority of human cancers, higher immune response is significantly associated with better clinical outcome (OS, PFI, or both), which is in agreement with previous reports [5, 7] that pre-existing immunity is probably necessary for most treatment response. More prominently, we demonstrated as well that in some cancer types higher immune response is significantly associated with worse outcome. This unexpected result indicates the diversity of mechanisms controlling antitumor immunity in different cancer types and suggests new strategies to promote the cancer immunity cycle. However, these survival results were obtained via retrospective analyses, and some cancer types had small patient sample sizes. Therefore, prospective confirmation or functional validation is needed to further corroborate these associations. Moreover, impacts of specific immune cell types on cancer prognosis warrant future investigation.

In summary, we have quantitatively characterized the pre-existing immune conditions in a wide spectrum of human cancers based on our recently identified immune gene expression signature and systematically examined the relationship of immune response and clinical outcome. Immune responses vary from cancer to cancer and have different associations with patient outcome in different human cancers.

## MATERIALS AND METHODS

### Patient samples

We previously described the global immune gene signature that we identified using TCGA PanCanAtlas patient gene expression data [14]. Patient survival data, including the OS time, PFI, and corresponding event status, were obtained from the TCGA PanCanAtlas Research Network [43]. A total of 10,062 PanCanAtlas tumor samples had both gene expression and survival data, covering 32 different cancer types. Data for patients with acute myeloid leukemia were excluded from this study because of a lack of clinical information.

### Spatial characteristics of the global immune gene signature

We annotated the immune gene signature, including the genes’ cellular locations, by using the Ingenuity Knowledge Base (Qiagen; https://www.qiagenbioinformatics.com). Information on available drugs targeting the genes in the signature was also retrieved from this annotation tool. We used Genome Reference Consortium human genome assembly GRCh38 to map the genomic coordinates of the immune gene signature to the human genome and then visualize the gene distribution in two-dimensional space, representing chromosomal positions. One dimension consisted of the 23 chromosomes from Chr 1 to Chr X, and the other dimension indicated the genomic coordinates on a chromosome from short (*p*) arm to long (*q*) arm.

### Gene expression analysis

To visualize the expression of the immune gene signature across human cancers, we next analyzed the mRNA expression profiles of the 382 immune genes in the signature using methods similar to those previously reported [14]. The gene expression data were first median centered across all the 10,062 PanCanAtlas tumors and then log transformed. Next, we used the next-generation clustered heat map (NG-CHM) tool developed at The University of Texas MD Anderson Cancer Center [44] to visualize the expression profiling of the immune gene signature among different cancer types. The patient samples in the heat map were ordered as follows. We first sorted the 32 cancer types in ascending order from left to right on the basis of their median immune response. Within each of the individual cancer types, patients were further sorted in ascending order based on their immune response.

### Immune metric generation

By using the global immune gene signature, we devised an mRNA-based immune metric to quantify the immune response of each tumor sample. In brief, we first calculated the *z* score of each gene of the 382 immune signature genes across all the PanCanAtlas samples. Then we took the median of all calculated *z* scores within this immune gene signature as the immune metric for a quantitative surrogate of anti-tumor immune response. By this approach, we calculated the immune response metric for all 10,062 PanCanAtlas tumor samples. These metrics can be used to quantify the pre-existing immune conditions of the tumor microenvironment. A similar approach has been successfully applied in our previous studies to characterize activities of the TGF-beta pathway [15] and tricarboxylic acid cycle metabolic pathway [18]. A much simpler approach involving only two genes was previously used to quantify immune cytolytic activity [20]; these two genes are included in the global immune gene signature.

### Survival analysis

Two clinical survival outcome endpoints were chosen for analysis of association with immune response, OS and PFI [43]. OS was defined as the interval from the date of initial diagnosis to the date of last known contact (censored) or death. PFI was defined as the period from the date of diagnosis until the date of the first occurrence of a new tumor event, which included progression of the disease, locoregional recurrence, distant metastasis, new primary tumor, or death with tumor. Patients who were alive without these event types or who died without tumor were censored. The event time was the shortest period from the date of initial diagnosis to the date of an event. The censored time was from the date of initial diagnosis to the date of last contact or the date of death without disease. Of note, the survival times (OS and PFI) varied with a wide range because multiple cancer types were included.

The Kaplan-Meier method was used to examine the association of tumor immune response with patient survival outcomes. For each cancer type, we first filtered out patients either with no survival data available (no survival time or event status) or with survival time equal to zero. The remaining patients were then dichotomized into two groups based on the median immune metric. Patients with an immune metric value greater than or equal to the cutoff were categorized into the high-immune-metric group, while those with an immune metric value less than the cutoff were categorized into the low-immune-metric group.

### Statistical analysis

The Kaplan-Meier method was used to evaluate survival difference between the dichotomic groups stratified by the immune response in each of the 32 cancer types. Statistical significance in survival difference was assessed via the Mantel-Cox log-rank test. All statistical tests were two-sided, and a *P* value of less than 0.05 was considered significant. The calculations and graphs were made with GraphPad Prism, version 7.03 (GraphPad Software, Inc., La Jolla, CA, USA).
